# On the Delivery of the Child by Turning, as a General Rule in Labor

**Published:** 1859-09

**Authors:** E. Garland Figg

**Affiliations:** Borrowstowness


### CURIOSITIES OF MEDICAL LITERATURE.

Under this head we purpose occasionally to chronicle some of the "sayings and doings" of members of the medical profession, of reputed sanity; not with a view to the sanction of unauthorized proceedings, but as a warning to others to avoid practices that can only result in disgrace and trouble.

*On the Delivery of the Child by Turning, as a General Rule in Labor. By Mr. E. Garland Figg, of Borrowstowness.* (Medical Times and Gazette, Ranking's Abstract.)

In this paper, Mr. Figg attempts to show, not only that delivery by turning is preferable to delivery by the forceps in cases requiring operative interference, but that turning is the rule to be adopted in general cases. He tells us that he has attended sixty labors since writing these papers; that only three of these were conducted as head presentations, and that of the remainder two were breech presentations, and fifty-five deliveries by turning. As the results of this astonishing practice, we leave Mr. Figg to speak for himself:

"With regard to the children, they are generally still from two to five minutes, and in some cases, half an hour's duration. In many instances, the first arm brought down is a little painful when moved, for a day or two. I confess with humility, I have even broken four arms, which, though they occurred in cases of great pelvic contraction, were attributable to my own mismanagement in pressing over the shaft of the os humeri instead of following its line to the elbow. Should you commit the same error, with similar result, be not too candid to the relatives, but at once by your own dictum transubstantiate the injury into a slight sprain received by the infant striking its shoulder against the backbone of the mother while actively prosecuting its uterine gambols. It will pass current, more especially if you appeal to her experience, when it is sure to be corroborated by a quotation and the day and hour of the occurrence. Two slips of pasteboard applied, with a strip of calico a yard long, remedies the evil in ten days. In establishing a comparison between the advantages derivable from turning in primiparæ and multiparæ, I believe there is a preponderance of argument in favor of the former. In a primapara the os uteri is more in the axis of the pelvic brim, the body of the organ being more inclined to the perpendicular, and not projecting anteriorly, as in the frequent parturient; hence, in the former case, the uterine efforts of the last month previously to labor lodge the os and cervix, inclusive of the head, low in the cavity of the pelvis, not only assuring the practitioner by tangible proof of the perfect capacity of the brim, but also presenting the best arrangement for the co-operation of the uterus with his extractive efforts. In the latter case, from the yielding of the abdominal muscles in former labors, the fundus bearing forwards throws the os in the direction of the spine, rather than the pelvic cavity. Hence, until the contraction of the muscles in some measure restores the proper axis, no advance can take place. The advantage in the second particular is briefly explained, by stating that in a primipara the antagonistic force is directly in line with the extractive. In a multipara it is entrenched round a corner. Again, in a primiparal case, you have good grounds for the conviction that in obviating the perineal stage, you limit the labor considerably; while in the latter patient an hour's suffering might conclude the case. Be they right or wrong, these are the sentiments that have guided my conduct in a large majority of my cases lately, experience appearing to justify in happy results what theory dictated in sound reasoning. I hope I shall soon lose all mental impressions of a head lingering on the perineum, or stationary from failing pains for hours. My primiparal patients are up in four days, without swelling of vaginal muscles, nymphæ or labia; *and what to me is perfectly unaccountable*, with very slight laceration of the perineum. I have had but one maternal death where the infant was turned, and that occurred five days after the event, by inflammation of the peritoneum of a patient, who with contracted pelvis had submitted to the ordeal to produce her sixth full-timed dead child. If I be entitled to any credit at all, it is for the candid avowal of a practice that some, under fear of professional censure, would have adhered to, but concealed."—*Ranking's Abstract.*

We find from statistics now before us, that out of 169 cases of turning, where the result to the mother is specially mentioned, 11 mothers died, or 1 in 15, and in 542 cases where the result to the child is detailed, 182 children were lost, or rather less than one in three. This is the result of the practice of Mad. Lachapelle, Mad. Boivin, Clark, Collins, Cusack, Gregory, Beatty, Churchill, etc. Their names are sufficient guarantee for skill. Mr. Figg states the children were generally still-born, some for "half an hour's duration," and as he tells us of no deaths amongst them, we infer he implies there were none amongst the sixty labors. But what is to us just as

"unaccountable" as to himself, if the children escape death, how the mothers that are primiparæ escape laceration of the perineum. We appeal to the experience of any midwifery practitioner, if some of the most anxious moments of his professional life have not been caused by cases where turning was necessarily resorted to in primiparæ, and where the chances of the infant's life and of the mother's perineum lay in the balance. Yet Mr. Figg proposes to manufacture recklessly—we may almost say unscrupulously—this very state of things, which to others so often involves a distressing alternative—either a dead child or a mutilated mother. He says his practice is based on "experience" and "sound reasoning." Quite the contrary; it is in direct opposition to the experience of the most successful practitioners, and utterly at variance with humanity, nature, and common sense. We therefore refuse to accept his conclusions, and after the sage advice he so conscientiously gives for deceiving the friends of our patients, he will excuse us in telling him that even his facts are open to suspicion.

